# A case of severe pseudohyperkalaemia due to muscle contraction

**DOI:** 10.11613/BM.2020.021004

**Published:** 2020-06-15

**Authors:** Jan Van Elslande, Toon Dominicus, Jaan Toelen, Glynis Frans, Pieter Vermeersch

**Affiliations:** 1 Clinical Department of Laboratory Medicine, University Hospitals Leuven, Leuven, Belgium; 2 Department of Pediatrics, University Hospitals Leuven, Leuven, Belgium; 3 Department of Development and Regeneration, University of Leuven, Leuven, Belgium; 4 Department of Cardiovascular Sciences, University of Leuven, Leuven, Belgium

**Keywords:** hyperkalaemia, pseudohyperkalaemia, preanalytical phase, case report

## Abstract

**Introduction:**

Severe hyperkalaemia is a serious medical condition requiring immediate medical attention. Before medical treatment is started, pseudohyperkalaemia has to be ruled out.

**Case description:**

A 10-month old infant presented to the emergency department with fever and coughing since 1 week. Routine venous blood testing revealed a severe hyperkalaemia of 6.9 mmol/L without any indication of haemolysis. Reanalysis of the plasma sample confirmed the hyperkalaemia (7.1 mmol/L). Based on these results, the clinical pathologist suggested to perform a venous blood gas analysis and electrocardiogram (ECG) which revealed a normal potassium of 3.7 mmol/L and normal ECG, ruling out a potentially life-treating hyperkalaemia. The child was diagnosed with pneumonia. The paediatrician had difficulty to perform the first venous blood collection due to excessive movement of the infant during venipuncture. The muscle contractions of the child in combination with venous stasis most probably led to a local increase of potassium in the sampled limbs. The second sample collected under optimal preanalytical circumstances had a normal potassium. Since muscle contraction typically does not cause severe hyperkalaemia, other causes of pseudohyperkalaemia were excluded. K_3_-EDTA contamination and familial hyperkalaemia were ruled out and the patient did not have extreme leucocytosis or thrombocytosis. By exclusion a diagnosis of pseudohyperkalaemia due to intense muscle movement and venous stasis was made.

**Conclusion:**

This case suggests that intense muscle contraction and venous stasis can cause severe pseudohyperkalemia without hemolysis. Once true hyperkalemia has been ruled out, a laboratory work-up can help identify the cause of pseudohyperkalaemia.

## Introduction

Pseudohyperkalaemia can be defined as a raised *in vitro* potassium concentration without an actual hyperkalaemia *in vivo* (the latter being called “true hyperkalaemia”). This situation can be caused by either a release of potassium from cells during or after phlebotomy or by contamination with potassium containing substances (*1*). Pseudohyperkalaemia is typically suspected in patients with an unexpected potassium result, who do not have any clinical symptoms (*e.g.* muscle weakness or paralysis, cardiac arrhythmia, electrocardiogram (ECG) abnormalities) or typical risk factors (*e.g.* cell lysis, renal failure, diabetic ketoacidosis, certain drugs) for true hyperkalaemia, a potentially lethal condition. Hyperkalaemia is defined as a serum potassium concentration exceeding 5.0 mmol/L. The cut-off to define a “severe hyperkalaemia” or “hyperkaliaemic emergency”, varies between 6.0 and 6.5 mmol/L (*2*-*4*). In some cases, pseudohyperkalaemia has even led to patients being unnecessarily haemodialyzed (*5*). We present a case of pseudohyperkalaemia mimicking severe hyperkalaemia in a child, in the absence of haemolysis.

## Case description

A 10-month old infant presented to the emergency department with fever and coughing since 1 week. The boy was born at 37 weeks postmenstrual age, had no relevant personal or familial antecedents, and did not take any medications at the time of admission. Informed consent was obtained from the parents. Clinical examination showed fever (38.2°C), a bilateral otitis media, and crepitations over the base of the right lung. There was no cardiorespiratory or metabolic distress and there was still adequate fluid and food intake and urine production. Routine venous blood testing showed inflammation with a high-normal white blood cell (WBC) count of 15.6 x10^9^/L (reference interval (RI): 6.0 - 17.5 x10^9^/L) and an increased CRP of 97.4 mg/L (RI: ≤5 mg/L) (Table 1). A chest X-ray showed a consolidation suspicious for pneumonia. Based on these findings the child was diagnosed with pneumonia and antibiotic treatment was started.

**Table 1:** Laboratory results

**Venous plasma sample (Roche Cobas 8000)**				**Venous whole blood sample (Radiometer ABL90 flex)**		
**Analyte (unit)**	**Original results** **(14:53 h)**	**Repeated results** **(15:56h)**	**Reference interval**	**Analyte (unit)**	**Results (16:00h)**	**Reference interval**
Potassium (mmol/L)	6.9	7.1	3.5 - 4.5	pH	7.44	7.35 - 7.43
Sodium (mmol/L)	137	138	136 - 145	pCO_2_ (kPa)	5.1	/
Chloride (mmol/L)	101	101	98 - 107	pO_2_ (kPa)	4.9	5.1 – 5.9
Urea (mmol/L)	2.0	/	≤ 8.2	O_2_ saturation (%)	70	70 - 80
Creatinine (µmol/L)	133	/	141 - 345	HCO_3_^-^ (mmol/L)	25.9	22.0 - 29.0
Calcium (mmol/L)	/	2.10	2.15 - 2.55	Base excess (mmol/L)	1.7	- 2.0 - 3.0
Magnesium (mmol/L)	/	0.54	0.63 - 1.05	Haemoglobin (g/L)	105	120 - 160
HCO_3_^-^ (mmol/L)	19	/	22 - 29	Haematocrit (%)	33.2	37.0 - 47.0
Total protein (g/L)	**/**	78	66 - 88	Sodium (mmol/L)	137	136 - 146
Albumin (g/L)	/	42	35 - 52	Potassium (mmol/L)	3.7	3.5 - 4.5
CRP (mg/L)	97.4	/	≤ 5.0	Chloride (mmol/L)	102	98–106
AST (U/L)	37	/	≤ 31	Anion gap	12.8	10.0 - 20.0
ALT (U/L)	17	/	≤ 31	Calcium ionized (mmol/L)	1.30	1.15 - 1.29
ALP (U/L)	221	/	122 - 469	Glucose (mmol/L)	6.7	3.1 – 5.6
Glucose (mmol/L)	4.8	/	3.1 – 5.6	Lactate (mmol/L)	1.4	0.5 - 2.2
Triglycerides (mmol/L)	/	1.0	< 1.7			
Cholesterol (mmol/L)	/	3.6	< 4.9			
Free haemoglobin (g/d)	0.10	0.14	/			
Icteria index	0	0	/			
Lipaemia Index	16	19	/			
**Venous whole blood sample (Sysmex XE 5000)**						
Haemoglobin (g/L)	105	/	110 - 135			
White blood cells (x10^9^/L)	15.6	/	6.0 - 17.5			
Red blood cells (x10^12^/L)	4.36	/	3.70 - 5.30			
Platelets (x10^9^/L)	630	/	150 - 450			
CRP – C-reactive protein. AST – aspartate aminotransferase. ALT – alanine aminotransferase. ALP – alkaline phosphatase. pO_2_ – partial pressure of oxygen. pCO_2_ – partial pressure of carbon dioxide. HCO_3-_ - bicarbonates.						

The routine venous blood draw did, however, also reveal a severe hyperkalaemia of 6.9 mmol/L (RI: 3.5 - 4.5 mmol/L) without any indication of haemolysis (estimated free haemoglobin: 0.1 g/L). A spurious hyperkalaemia was suspected since there were no apparent risk factors or symptoms for true hyperkalaemia. The clinical pathologist suggested to perform an ECG and a blood gas analysis to rule out a true hyperkalaemia. The blood gas analysis of a new venous sample drawn 67 minutes after the first sample, revealed a normal potassium of 3.7 mmol/L. The ECG was also normal and the patient showed no other clinical signs of hyperkalaemia.

The paediatrician stated that the initial blood draw had been very difficult in this patient with part of the blood collected from the forearm and part from the foot. The child resisted by repeatedly trying to move his arms and legs, requiring two nurses to restrain the child. A tourniquet was used to create venous stasis. The control blood sample for blood gas analysis, in contrast, was an easy and quick collection from the contralateral forearm.

## Laboratory analyses and other diagnostic evaluations

The original venous blood samples were collected using a Neoflon Pro 24G catheter (Becton Dickinson, Temse, Belgium) in a 1 mL Lithium Heparin MiniCollect tube (Greiner Bio-One GmbH, Kremsmünster, Austria) and a 1.3 mL K_3_- ethylenediaminetetraacetic acid (EDTA) Micro tube (Sarstedt, Nümbrecht, Germany). The initial samples were sent to the laboratory *via* pneumatic tube system and cells were separated from plasma within 30 minutes after collection. The lithium heparin sample was centrifuged for 10 minutes at 2000xg. Routine chemistry tests were measured on a Roche Cobas 8000 ion-selective electrode (ISE) module and a c702 module (Roche Diagnostics, Rotkreuz, Switzerland). Haemoglobin and cell counts were performed using a XE-5000 cell counter (Sysmex GmbH, Norderstedt, Germany). The venous blood sample for blood gas analysis was collected 67 minutes later using a 1 mL heparinized blood gas syringe (Westmed, Tucson, Arizona) and analysed in the emergency department within 5 minutes after collection on a Radiometer ABL90 FLEX blood gas analyzer (Radiometer, Kopenhagen, Denmark).

After true hyperkalaemia was excluded based on the normal ECG and normal potassium on blood gas analysis, possible causes of pseudohyperkalaemia were systematically investigated (Table 2). Reanalysis of the initial plasma sample (stored at 4°C for 1 hour after separation of cells) confirmed the hyperkalaemia (7.1 mmol/L) and the absence of haemolysis (estimated free haemoglobin: 0.14 g/L), excluding haemolysis during blood collection or transport via the pneumatic tube system. Internal quality control results were normal and no abnormalities were noted in other patient samples analysed on the same ISE-module, ruling out a technical problem. The WBC count was high-normal with a normal cell differential (data not shown), excluding pseudohyperkalaemia due to leucocytosis, which can occur when WBC > 50 x10^9^/L (*6*). The platelet count was elevated to 630 x10^9^/L (RI: 150 – 450 x10^9^/L), but this was ruled out as cause of the spurious elevation as there was no sample clotting (heparinized plasma) (*6*). Transport time was short, as mentioned earlier (< 30 minutes), and the sample was not cooled during transport. The child did not receive any potassium containing infusions. The order of blood draw was verified with the clinician who confirmed that the heparin sample was drawn before the K_3_-EDTA sample. K_3_-EDTA contamination was also unlikely since an open system (blood dripping into open tubes) was used for blood collection and calcium, magnesium and alkaline phosphatase activity were normal (*7*).

**Table 2:** Possible causes of spurious hyperkalaemia and investigations

**Release from cells**			
**Origin of potassium**	**Investigations**		**Examples of aetiologies**
In vitro haemolysis (cell disruption)	H-index		Small gauge needle, use of vacuum tube Rough transport conditions (*e.g.* freezing whole blood, pneumatic tube system)
Release from RBC (no cell disruption)	Check transport conditions Screening experiment: centrifugation after several hours *vs.* immediate centrifugation		Cold temperature (*e.g.* refrigeration) Familial pseudohyperkalaemia Hereditary stomato-, xero- or spherocytosis
Release from platelets (during clotting)	Thrombocyte count > 500 x10^9^/L Serum *vs.* plasma sample		Reactive thrombocytosis Myeloproliferative disorders
Release from WBC (cell disruption)	WBC > 50 x10^9^/L Cell differential		Reactive leucocytosis Chronic lymphocytic leukaemia Acute leukaemia
Release from muscle cells	Inquire about blood draw conditions		Prolonged venous stasis Fist clenching/pumping
**Contamination with potassium-rich fluids**			
K_2_-EDTA or K_3_-EDTA		Hypocalcaemia, hypomagnesemia, decreased alkaline phosphatase activity Inquire how blood collection was performed (Measure EDTA)*	Order of draw not followed correctly Decanting of tubes
Infusion		Check medical file	K-penicillin KCl infusion
RBC – red blood cells. WBC – white blood cells. EDTA – ethylenediaminetetraacetic acid. KCl - Potassium chloride. H – haemolysis. Information based on (10), (18), (19) and (21). *This test is not routinely available in most laboratories.			

## Considered diagnoses and further investigations

Since other causes of pseudohyperkalaemia had been excluded, familial pseudohyperkalaemia and pseudohyperkalaemia due to intense muscle contraction and venous stasis were taken into consideration (Table 2). In familial pseudohyperkalaemia, a rise in potassium is expected when sample processing is delayed due to increased potassium leakage through the red blood cell membranes (*8*). To exclude this cause, a new heparinized plasma sample was requested and split into two aliquots upon arrival in the laboratory. This sample was collected two days later before the patient was discharged from the hospital. One aliquot was immediately centrifuged while the other was kept at room temperature for two hours before centrifugation. Iolascon *et al.* who identified the genetic locus for familial pseudohyperkalaemia due to red cell leakage observed an increase of 1 mmol/L after 2 hours storage at room temperature in 3 patients affected by this disorder (*9*). The difference between both aliquots in our patient was negligible (4.7 vs. 4.6 mmol/L = fractional increment of - 2%), thereby excluding familial pseudohyperkalaemia.

Since all other causes of pseudohyperkalaemia were ruled out, we concluded that the spurious hyperkalaemia in our patient was most likely due to the intense muscle contraction and venous stasis during blood collection.

## What happened?

During the collection of the first venous blood sample (at hospital admission), the muscle contractions of the child, in combination with venous stasis (by application of tourniquet and manual force by the nurses) most probably led to a local increase of the potassium concentration in the sampled limbs. When the tourniquet was removed, the blood from the sampled limbs was diluted in the systemic blood circulation without causing clinical symptoms of hyperkalaemia. However, in the venous blood sample sent to the laboratory the potassium concentration was increased. This finding is *strictu sensu* not “spurious” since the local level of potassium in the limb is truly elevated *in vivo*. We did, however, consider this increase as “spurious” or “pseudo” because the laboratory result does not reflect the potassium concentration in the systemic circulation of the patient. The unexpected result triggered an additional blood gas measurement to rule out a potential false increase of potassium. Potassium was normal in this sample which was collected under optimal preanalytical circumstances. This result can be considered as representative for the systemic potassium concentration.

## Discussion

This case report suggests that intense muscle movement and venous stasis can cause a spurious hyperkalaemia with a potassium > 6.5 mmol/L, mimicking a severe true hyperkalaemia. The clinician and pathologist suspected a spurious potassium result since there were no clinical symptoms of hyperkalaemia. The ethology of this spurious elevation was suspected to be muscle movement combined with venous stasis only after exclusion of other (more common) causes of pseudohyperkalaemia.

It is well known that prolonged venous stasis during venipuncture and muscle contraction (*e.g.* fist clenching) can lead to increases in potassium concentration through release by muscle cells, but the increase in the limb confined by venous stasis typically does not exceed 2.0 mmol/L (*10*-*13*). In an experimental study where volunteers were asked to perform 10-15 rhythmic contractions of the forearm after application of a pressure cuff, elevations of up to 1.7 mmol/L were noted when blood was collected via a superficial vein. During application and after release of the pressure cuff, no hyperkalaemia was observed in samples obtained from the contra-lateral arm, indicating that the potassium level in the systemic circulation did not elevate significantly (*10*). In this case, the infant did not just clench his fists, but extensively contracted his arm and leg muscles, which could explain the large increase in potassium of 3.2 mmol/L.

In vitro haemolysis during and after blood collection is the most common cause of spurious hyperkalaemia with a prevalence of up to 3.3% of routine samples (*14*). The low free haemoglobin concentration in this case (0.1 g/L) excluded both haemolysis that can occur during blood collection (*e.g*. by using a fine needle) and after blood collection (*e.g.* by cell lysis during pneumatic tube transport).

Potassium EDTA (K_2_-EDTA or K_3_-EDTA) contamination is also a common cause of spurious hyperkalaemia (*15*). White *et al.* found EDTA contamination, defined as > 0.1 mmol/L EDTA, in 0.5% (N = 22/4789) of samples (hyperkaliaemic and non-hyperkaliaemic) received for routine analysis (*16*). In our laboratory, measurement of EDTA is not available. We therefore excluded this cause of the spurious hyperkalaemia by assessing the concentrations of calcium and magnesium. A spurious elevation in potassium concentration of 3.0 mmol/L would require a contamination with 1.5 mmol/L EDTA for K_2_-EDTA or 1.0 mmol/L EDTA for K_3_-EDTA. Since EDTA plasma contains approximately 10.3 mmol/L EDTA for a haematocrit of 0.400 L/L (6.16 mmol or 1.8 g EDTA *per* litre of whole blood, as stated by the manufacturer), an increase of 3 mmol/L would correspond to a 10% contamination with K_3_-EDTA plasma or 15% for K_2_-EDTA plasma. Such a level of contamination has been experimentally shown by Lima-Oliveira *et al.* to systematically result in hypocalcaemia and hypomagnesemia (*17*). The absence of hypocalcaemia and hypomagnesemia in the initial venous blood sample of the child argues against EDTA contamination as cause of the spurious hyperkalaemia.

Familial pseudohyperkalaemia was also considered in this case, since this can also result in spurious hyperkalaemia without haemolysis (*18*). This can be caused by a mutation in the ABCB6 transporter (formerly called “leaky cell membrane syndrome”) and by several red blood cell membrane defects such as hereditary stomato-, xero- or spherocytosis (*18*, *19*). We did, however, not find any increase after storage for 2 hours at room temperature, excluding familial pseudohyperkalaemia as cause of the spurious hyperkalaemia.

A blood gas analysis can help to quickly distinguish a true hyperkalaemia, a potentially lethal condition, from a spurious hyperkalaemia. The advantage of a bedside blood gas analysis is that it is not susceptible to a spurious increase in potassium concentration due to leucocytosis, thrombocytosis or familial pseudohyperkalaemia. Blood gas analysis is, however, not immune to preanalytical errors leading to spurious hyperkalaemia such as in vitro haemolysis and EDTA contamination as illustrated by a recent case report by Salvagno *et al.* who described spurious hyperkalaemia due to haemolysis in a blood gas sample which was put on ice (*20*).

A limitation of our case report is that the evidence for pseudohyperkalaemia due to muscle contraction and venous stasis is circumstantial and that this remains a diagnosis by exclusion. Another limitation is the fact that we did not measure EDTA in the serum samples to confirm the absence of EDTA contamination, although we were able to indirectly rule out EDTA contamination based on the results of potassium, calcium and magnesium.

In conclusion, a severe spurious hyperkalaemia is presented in this case report, most likely caused by intense muscle contraction combined with venous stasis. This “false” increase of 3.2 mmol/L is higher than what has previously been described in the literature.

## What you can do in your laboratory to prevent such errors

Unexplained high potassium results should always be discussed between the laboratory specialist and the requesting physician to avoid unnecessary potassium-lowering treatment due to spurious hyperkalaemia.
A bedside blood gas analysis performed under optimal preanalytical conditions can help to quickly distinguish true hyperkalaemia from a spurious increase.
